# Conceptual framework for support of caregivers of children diagnosed with intellectual disabilities in Gauteng

**DOI:** 10.4102/curationis.v45i1.2316

**Published:** 2022-10-28

**Authors:** Lebogang L. Molefe, Leepile A. Sehularo, Daleen M. Koen

**Affiliations:** 1 Department of Nursing, Faculty of Health Science, North-West University, Mahikeng, South Africa

**Keywords:** conceptual framework, support, caregiver, children, diagnosed, intellectual, disabilities

## Abstract

**Background:**

Caregivers of children diagnosed with intellectual disabilities in the Gauteng province face numerous challenges while caring for children. Their needs are not met. There is lack of support for caregivers and no existing conceptual framework of support. There was therefore a need to develop a conceptual framework of support for caregivers.

**Objectives:**

The purpose of this study was to develop a conceptual framework of support for caregivers of children diagnosed with intellectual disabilities in the Gauteng province.

**Method:**

Three phases were followed: an empirical phase, a classification of concepts and a development phase. A conceptual framework was developed based on the results of the empirical phase, after concepts were classified.

**Results:**

The conceptual framework of support for caregivers of children diagnosed with intellectual disabilities in the Gauteng province was developed, consisting of six components, namely agents, recipients, context, procedures, dynamics and terminus.

**Conclusion:**

A framework will guide all stakeholders on how to support caregivers of children diagnosed with intellectual disabilities in the Gauteng province.

**Contribution:**

The framework serves as a guide for future studies aiming at developing support programmes and models for caregivers, and further provide guidance on how caregivers can effectively be supported when used in mental healthcare institutions.

## Introduction

The focus of this study is on a conceptual framework for support of caregivers of children diagnosed with intellectual disabilities in the Gauteng province. A conceptual framework is a visual or written product that explains, either graphically or in a narrative form, the main components to be studied, the key factors, concepts or variables and the presumed relationships among them (Ruseckaite et al. 2019:357). According to Polit and Beck (2014:123), a conceptual framework describes the strategic objectives in association with an action plan to achieve the research aim. A conceptual framework, as defined by Regoniel (2015:1), serves as a ‘map’ or ‘rudder’ that will guide one towards realising one’s own study’s objectives or intent and represents the researcher’s synthesis of the literature on how to explain a phenomenon. According to Regoniel (2015:2), a conceptual framework maps out the actions required in the course of the study, given previous knowledge of other researchers’ ‘point of view and observations on the subject of research’.

The authors therefore used previous knowledge of other researchers’ point of view, obtained from a systematic review conducted on support needed by caregivers of children diagnosed with intellectual disabilities and semistructured interviews with caregivers on challenges and needs of caregivers of children diagnosed with intellectual disabilities in the Gauteng province. In addition, semistructured interviews were also conducted with mental healthcare practitioners on perceptions of mental healthcare practitioners regarding the support for caregivers of children diagnosed with intellectual disabilities in the Gauteng province. A conceptual framework for this study is therefore based on the concepts that emerged from results of the three studies. According to the Diagnostic and Statistical Manual for Mental Disorders (DSM-5), intellectual disability is defined as a neurodevelopment disorder characterised by impairment in general abilities that impact adaptive functioning in conceptual, social and practical domains. A person with intellectual disability has an intelligence quotient (IQ) of about 70 or below. The DSM-5 further emphasises the need to use both clinical assessment and standardised testing of intelligence when diagnosing intellectual disability, with the severity of impairment based on adaptive functioning rather than IQ test score alone (American Psychiatric Association 2013:145). Caregivers are explained by Jegermalm and Torgé (2021:7) as people whose occupation involves providing a face-to-face service that develops the human capabilities of the recipient. Human capabilities means health, skills or proclivities that are useful to oneself or others. These include physical and mental health, physical skills, cognitive skills and emotional skills (Razavi & Staab 2010:408). In the South African context, a caregiver tends to the needs of a person with short- or long-term limitations because of illness, injury or disability, whether in home healthcare, assisted living facilities, nursing homes or day care centres (Ayob, Christopher & Naidoo 2021:86). Caregivers therefore assist with personal care such as bathing and grooming, dressing, toileting, stimulations, exercises and overseeing medication usage. To be a registered caregiver in South Africa, the service provider that employs a person must ensure that they undergo or have undergone home-based care training and have a certificate that indicates that they received the training from an institution accredited by the South African Qualification Authority (SAQA). A diploma or any other highest qualification is not necessarily a requirement.

It is therefore evident that the work that caregivers do is very important and therefore needs to be appreciated and supported at all times. Globally, including the Gauteng province, psychological morbidity of caregivers is often ignored, and this affects both the quality of care delivered to children and their professional as well as their personal lives (Cottagiri & Sykes 2019:175; Maphosa & Ciwanza 2021:157; Ormel et al. 2017:72; Penson et al. 2018:428; Zuurmond et al. 2019:45).

The study by Gerain and Zech (2019:2) affirms that caregivers’ well-being is often ignored, because caregivers reported emotional exhaustion, burnout, depersonalisation and personal difficulties associated with caring. Sallim et al. (2015:1036) also confirms that being a caregiver puts a person at risk of poorer mental and physical health. Another study by Revenson et al. (2016:24) indicates that being a caregiver can represent an experience that puts an individual under stress. Another study by Herman (2009:345) established that in the Gauteng province, caregivers often receive services of mental healthcare practitioners such as social workers, psychologists and counsellors because of stress and burnout associated with caring.

According to Collins, Murphy and Bierman (2014:189), each individual’s experience is unique; therefore, frameworks should consider each individual’s extent of experience and exposure, because ignoring the extent of exposure and experience of an individual will deem the framework ineffective.

## Problem statement

There is a lack of support for caregivers of children diagnosed with intellectual disabilities in the Gauteng province; hence, caregivers are often referred to mental healthcare practitioners because of stress and burnouts associated with caring (Herman 2009:340). The study by Herman (2009:340) in partnership and consultation with the National Department of Health, South Africa, and South African Stress and Health (SASH) confirms that caregivers are often referred to psychologists, social workers and counsellors. Literature reviewed for this study revealed no existing conceptual framework of support for caregivers of children diagnosed with intellectual disabilities in the Gauteng province. There is therefore a need to develop a conceptual framework of support for caregivers of children diagnosed with intellectual disabilities in the Gauteng province. The conceptual framework will not only benefit caregivers in the Gauteng province but could be applied to other provinces of the country and globally because where there is evidence of a conceptual framework for caregivers, such a framework would be focusing on one challenge without actually addressing numerous challenges that caregivers encounter, thus providing one and minimal intervention to improve the health of caregivers (Collins et al. 2014:189). For the framework to be rated to be effective in improving the health of caregivers, it must be able to address multiple challenges that caregivers encounter, challenges that negatively affect the holistic health of caregivers (Wight et al. 2016:23).

## Purpose of the study

The purpose of this study was to develop a conceptual framework of support for caregivers of children diagnosed with intellectual disabilities in the Gauteng province.

## Research method and design

### Design

A qualitative, exploratory, descriptive and contextual research design was used. The design allowed the researchers to explore a topic with limited coverage within the literature. It further allowed the participants of the study to contribute to the development of new knowledge. Prior to this study, little was known about various challenges that are experienced by caregivers, as well as effective interventions to such challenges (Hunter, McCallum & Howes 2019:2); hence, the researchers affirm that the design assisted in understanding of experiences of participants as lived in real life (Burns & Grove 2016:747). The design allowed the researchers to observe, describe and document aspects as they naturally occurred (Polit & Beck 2014:226). Three phases were followed: an empirical phase, a classification of concepts and a development phase. The three phases are explained next.

### Phase one: Empirical phase

This phase had two stages, systematic review and qualitative research methods.

#### Stage 1: Systematic review

A systematic review was conducted to gather information on existing content on support needed globally by caregivers of children diagnosed with intellectual disabilities, using five steps: formulating a clear review question, gathering and classifying data, performing critical appraisal, summarising the evidence and discussion. Relevant studies were searched in the databases, namely Google Scholar, ScienceDirect, Africa Journal, Emerald Insight, JSTOR, Boloka and EbscoHost. Critical appraisal was also performed to determine validity, reliability and applicability of information. Results were also reviewed by an independent reviewer.

**Results of systematic review:** Results revealed two themes: challenges experienced by caregivers, as well as support needed by caregivers.

#### Stage 2: Qualitative research method

Two sets of interviews were performed: one with caregivers and the other one with mental healthcare practitioners.

### Research instrument

Semistructured interviews were used to explore and describe challenges and needs of caregivers of children diagnosed with intellectual disabilities, as well as to explore perceptions of mental healthcare practitioners regarding the support for caregivers of children diagnosed with intellectual disabilities. Open-ended questions were used during interviews. The following questions were asked to the caregivers:

- How is it for you to take care of an intellectually disabled child?
- What did you experience taking care of an intellectually disabled child?
- How can you be supported in taking care of an intellectually disabled child?

The following questions were asked to mental healthcare practitioners:

- What are your responsibilities in caring for caregivers of children diagnosed with intellectual disabilities?
- What are your perceptions regarding caring for children with intellectual disabilities?
- In your opinion, how could caregivers be supported?
- Is there anything else that you would like to add?

### Setting

The study was conducted in six different mental healthcare institutions catering for children diagnosed with intellectual disabilities in the Gauteng province. The Gauteng province is the smallest urban province in South Africa. There are many informal settlements around the province; hence, many people are unable to afford the urban lifestyle and basic necessities such as clean water and sanitation. Unemployment and poverty in those areas is very high; hence, there are many people who are unable to afford basic necessities, including necessities required to adequately care for children diagnosed with intellectual disabilities. The province has three metropolitan municipalities that are not far from each other: Johannesburg metropolitan municipality, Tshwane metropolitan municipality and Ekurhuleni metropolitan municipality. Three mental healthcare institutions in Johannesburg metropolitan municipality were used. The first institution, the biggest of them all, had 58 children with intellectual disabilities and only 20 caregivers. The second one had 40 children and 18 caregivers. The third institution had 24 children and 10 caregivers. In Tshwane metropolitan municipality, only one governmental institution was identified, having 22 children and eight caregivers. At Ekurhuleni metropolitan municipality, there were two institutions. The first institution had 14 children and six caregivers and the second one had 20 children and eight caregivers. Therefore, total of six institutions were used for this study. All institutions had children with mild, moderate, severe and profound intellectual disabilities.

### Population and sampling

The study population comprised caregivers of children diagnosed with intellectual disabilities in the Gauteng province, as well as mental healthcare practitioners in Gauteng province. Participants were all from six different mental healthcare institutions in the Gauteng province. A purposive, nonprobability sampling technique was used to select participants. It involved conscious selection of participants with specific reference to cases from which one can learn a great deal about the central focus or purpose of the study. It is the best method to gain insight into a new area of study or to obtain in-depth understanding of a complex experience or event (Burns & Grove 2016:353). Twenty caregivers and 15 mental healthcare practitioners who voluntarily agreed to participate in the study were interviewed. Inclusive and exclusive criteria for the two groups of participants are explained next.

Inclusion criteria:

- All caregivers of children diagnosed with intellectual disability in the three identified municipalities of the Gauteng province, within six identified mental health institutions, were included in this study. Caregivers were known to the facility and have provided care for these children for no less than two years. All caregivers were over 18 years of age, and therefore they managed to give voluntary consent to participate. The caregivers were providing care to children under the age of 18 years. All caregivers who reside in the Gauteng province, whether because of work or because it is their permanent residence, were included.
- Mental healthcare practitioners working in the six identified mental health institutions within three municipalities in Gauteng. These are psychologists, psychotherapists, counsellors and social workers who often see caregivers during referrals. They had at least two or more than two years’ experience of working with caregivers of children diagnosed with intellectual disabilities.
- All participants were willing to communicate in English, Sotho and/or Tswana because a majority of people in the Gauteng province speak one of these languages.

Exclusion criteria were caregivers of children diagnosed with intellectual disabilities who have formed part of the case load of the researcher. This measure was to prevent bias.

### Data collection

Recruitment of participants was carried out by an independent practitioner after the principal researcher explained the study purpose, risks and benefits to participants. The researcher opted to utilise an independent practitioner during the recruitment process in order to avoid bias. Data were collected by the principal researcher using semistructured interviews. Caregivers were interviewed in September 2021, whereas mental healthcare practitioners were interviewed in October 2021 and November 2021. All interviews did not last for more than 1 h. All interviews were audio-taped as per agreement between the researcher and participants. Permissions to audiotape interviews were obtained and participants signed voluntary consent forms.

### Data analysis

Data analysis was carried out using content analysis and ATLAS.ti (ATLAS.ti Scientific Software Development GmbH, Berlin, Germany). Data analysis involves reducing data by the process of ‘coding’ and breaking down data into smaller units, which were then be organised into categories and themes (Aveyard & Goodman 2017:405). Firstly, the principal researcher transcribed data verbatim. Secondly, the researcher developed a coding scheme so as to code data. Conceptual categories were created, wherein important concepts that emerged were given labels. Thirdly, data were coded into themes and subthemes. Lastly, data were organised in a cohesive manner. A co-coder independently analysed transcribed data and developed his own themes and subthemes. Following independent data analysis by the researcher and co-coder, a meeting was convened between the two to discuss the findings. Where there were disagreements on themes and subthemes, a discussion was held and consensus on the final themes and subthemes was reached.

### Ethical considerations

The principal researcher obtained an ethical clearance certificate from the Health Research Ethics Committee of the North-West University (reference number NWU-00462-20-A1) to conduct this study. The principal researcher and co-authors of the study attended an ethics workshop to ensure understanding of research ethics and adherence to boundaries pertaining to research through conducting themselves in an ethical manner throughout the process of the study. All sources used were duly acknowledged. Participants’ rights were protected by ensuring that human rights took precedence over research objectives; hence, the researcher ensured that she did not expose participants to COVID-19 infections by adhering to preventive measures for spread of infection as outlined by the National Department of Health, the National Institute for Communicable Diseases (NICD), the World Health Organization (WHO) and North-West University (NWU). During data collection, the researcher took her own temperature and that of each participant, and all were below 37.5 °C. If the temperature had been found to be above 37.5 °C, the researcher would not have commenced the interviews. The researcher constantly observed the participants for signs of discomfort; there was no discomfort shown. If there had been signs of discomfort, the researcher would have stopped the interview immediately, performed a spot debriefing and referred the participant to a professional counsellor or a psychologist.

### Trustworthiness

Five testing criteria for trustworthiness were observed: credibility, dependability, confirmability, transferability and authenticity (Polit & Beck 2018:295). Credibility was attained by ensuring that the study was carried out in a way that enhanced the believability of the findings; hence, two types of data collection methods were used, systematic review and semistructured individual interviews. This also assisted to ensure that credibility is demonstrated to external readers. Dependability was attained through description and application of methodology, audit trial, triangulation and the use of a co-coder to verify the findings. Confirmability was attained through ensuring that the findings were not shaped by researcher’s bias, motivation or interest; hence, the researcher ensured that the data represent the information participants provided as well as accurate results of a systematic review conducted. Transferability was attained through thick description of the research methodology. The study will be published with sufficient descriptive data that can be evaluated by readers for purposes of applicability in different contexts. This will enable someone interested in making a transfer to reach a conclusion about whether transfer can be contemplated as a possibility. Authenticity was attained through ensuring that the report conveys the feeling of participants’ lives as they are lived, as well as accurate findings of a systematic review. This will ensure that readers are able to understand the lives being portrayed, with some sense of mood, experience, language and context.

## Discussion of results

Results of the interviews are divided into two aspects: results of caregivers and results of mental healthcare practitioners. These results are explained next.

### Caregivers’ results

Two themes emerged: challenges experienced by caregivers and needs of caregivers. A lack of training and specialised knowledge on intellectual disability was cited as a first challenge: ‘We did not go to psychiatry training, but we are expected to be competent in providing care for these children’ (P-J, female, 31 years old, secondary caregiver, titled careworker, with 7 years’ experience of caring). A lack of resources was cited as a second challenge: ‘There are no nappies, toiletries, sheets and blankets. Wheelchairs are not fit for children’ (P-C, female, 54 years old, secondary caregiver, titled careworker, with 8 years’ experience of caring). The third challenge cited was staff shortage, which leads to poor quality of care: ‘Staff shortage makes us unable to render quality care’ (P-K, female, 31 years old, secondary caregiver, titled careworker, with 10 years’ experience of caring). The fourth challenge was emotional trauma, which is the result of stress experienced by participants. The stress is caused by the negative manner in which managers treat participants: ‘Managers are stressing us’ (P-N, female, 33 years old, secondary caregiver, titled careworker, with 9 years’ experience of caring). The fifth challenge cited by participants was poor quality of care for children: ‘There is lack of proper food for children’ (P-C, female, 54 years old, secondary caregiver, titled careworker, with 8 years’ experience of caring). Poor management style was found to be sixth challenge: ‘Communication by managers is very bad’ (P-G, female, 33 years old, secondary caregiver, titled careworker, with 7 years’ experience of caring).

Furthermore, needs were cited by participants. The first need was the need for training on intellectual disability: ‘I need them to send us for training on intellectual disability’ (P-A, female, 52 years old, secondary caregiver, titled careworker, with 10 years’ experience of caring). Participants strongly felt that training would benefit them enormously. They said that training would empower them on how to care for children and enable them to provide quality care: ‘We want to learn more about intellectual disability and how to take care of children’ (P-I, female, 28 years old, secondary caregiver, titled careworker, with 5 years’ experience of caring); ‘We must be send to school for knowledge improvement’ (P-K, female, 31 years old, secondary caregiver, titled professional nurse, with 10 years’ experience of caring); ‘Training will give me tricks on how to handle children’ (P-T, female, 43 years old, secondary caregiver, titled careworker, with 12 years’ experience of caring). The second need cited was the need for staff increase: ‘We request more staff so that we have extra hands to care’ (P-A, female, 52 years old, secondary caregiver, titled careworker, with 10 years’ experience of caring). The third need was a need for improvement in communication by managers: ‘I wish management can speak to us more’ (P-G, female, 33 years old, secondary caregiver, titled careworker, with 7 years’ experience of caring). The fourth need was a need for salaries increase: ‘They must increase our salaries and give us bonuses’ (P-Q, female, 32 years old, secondary caregiver, titled careworker, with 7 years’ experience of caring). The last need was a need for emotional support: ‘I wish the department can make programmes that address our concerns wherein our emotional well-being is catered for; they must reassure us’ (P-J, female, 31 years old, secondary caregiver, titled careworker, with 7 years’ experience of caring).

### Mental healthcare practitioners’ results

Three themes emerged: provision of training, provision of support and provision of resources. Mental healthcare practitioners cited that there is a need for specialised trainings and in-service trainings for caregivers: ‘Caregivers need that special training’ (P-EE, female, 48 years old, social worker, with 20 years’ experience of social work). Furthermore, the need for emotional support for caregivers was recommended: ‘Caregivers need a lot of emotional support’ (P-CC, female, 47 years old, social worker, with 18 years’ experience of social work). Financial support was also cited as a necessity: ‘Caregivers need to be remunerated better because they work a lot!’ (P-KK, female, 34 years old, social worker, with 11 years’ experience of social work). There was a need for caregivers to have group therapy sessions as a means to manage work-related stress: ‘Groups should be formed where they do activities such as fun games to reduce stress’ (P-GG, male, 34 years old, psychotherapist, with 11 years’ experience of psychotherapy). There was a need for provision of equipment to care for children, adequate resources and financial resources: ‘Resources need to be looked at because they are struggling’ (P-II, female, 31 years old, psychologist, with 7 years’ experience of psychology). This sentiment was shared: ‘There is a need to get more staff, because this job is heavy’ (P-LL, male, 33 years old, social worker, with 10 years’ experience of social work). Furthermore, P-MM said, ‘There is a need for adequate funding’ (female, 38 years old, psychologist, with 12 years’ experience of psychology).

### Phase two: Classification of concepts

The aim of the study was to develop a conceptual framework of support for caregivers of children diagnosed with intellectual disabilities in the Gauteng province, guided by Swaen (2015:142). In developing a conceptual framework, the researcher had to classify concepts. During concept classification, the results of the empirical phase (systematic review, caregivers’ challenges and needs and mental healthcare practitioners’ perceptions) were used. Manuscripts for the three have been submitted for publication (Molefe et al. 2021a, 2021b, 2021c, unpublished). As per the previous explanation, the results of the systematic review revealed two themes: challenges experienced by caregivers and support needed by caregivers (Molefe et al. 2021a, unpublished). Results of caregivers revealed two themes: challenges experienced by caregivers and needs of caregivers (Molefe et al. 2021b, unpublished). Results of mental healthcare practitioners revealed three themes: provision of training, provision of support and provision of resources (Molefe et al. 2021c, unpublished). Classification of concepts was performed using Dickoff et al.’s (1968:422) six steps as outlined in Table 1.

**TABLE 1:** Steps of conceptual framework development.

Steps	Explanation of steps	Who or what are those?
Step 1: Agency	Who or what performs the activity	People who will implement the framework
Step 2: Recipient	Who or what receives the activity	People who will receive the framework
Step 3: Context	In what context is the activity performed	This is a place where the framework will be implemented
Step 4: Procedure	The guiding procedure, technique or protocol of the activity	This involves the path or steps to be taken when implementing the framework
Step 5: Dynamics	Power sources such as agents, recipients and context to ensure success of a framework	It includes activities to be performed to ensure success of the framework
Step 6: Terminus	The end point or accomplishment of activity	This is the end product or outcome of the framework

*Source*: Dickoff, J., James, P. & Wiedenbach, E., 1968, ‘Theory in a practice discipline: Practice oriented theory’, *Nursing Research* 17(5), 415–434. https://doi.org/10.1097/00006199-196809000-00006

**FIGURE 1:**
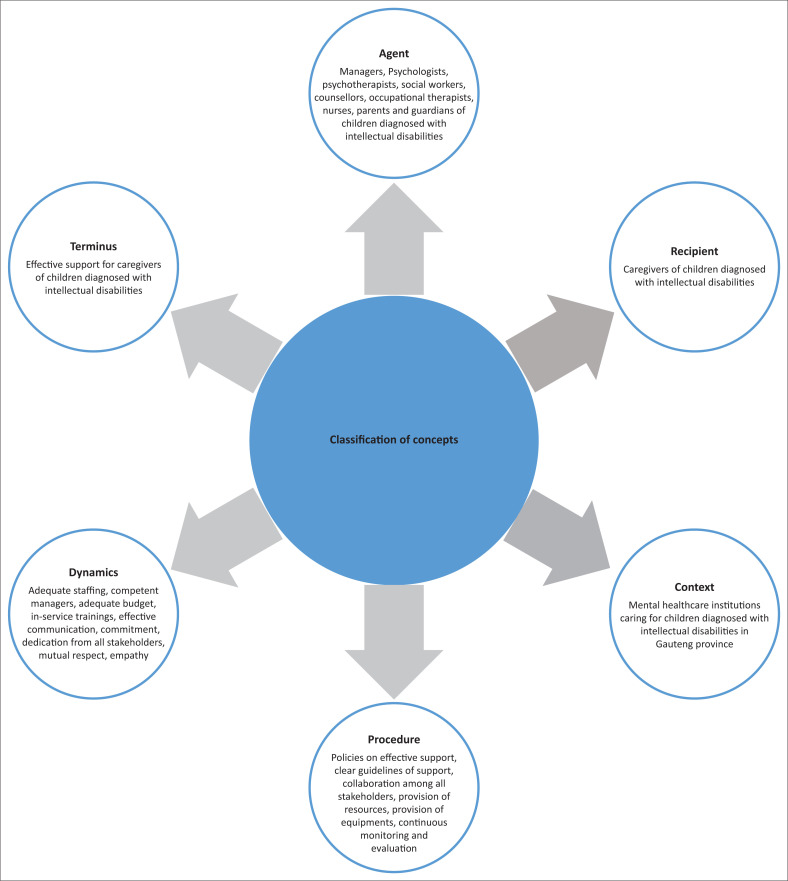
Concept classification, utilising steps of Dickoff et al. (1968:422).

According to Jabareen (2009:53) concepts of the framework should effectively represent the relevant social, cultural, political and environmental phenomenon or social behaviour and the multidisciplinary literature that focuses on the phenomenon under study and should come from a variety of types, such as books, articles, newspapers, essays, interviews and practices. It is for this reason that the researcher utilised concepts obtained from the empirical phase results. Figure 1 summarises concept classification, using Dickoff et al. (1968:422).

### Phase three: Development of the conceptual framework to support caregivers of children diagnosed with intellectual disabilities in the Gauteng province

**FIGURE 2:**
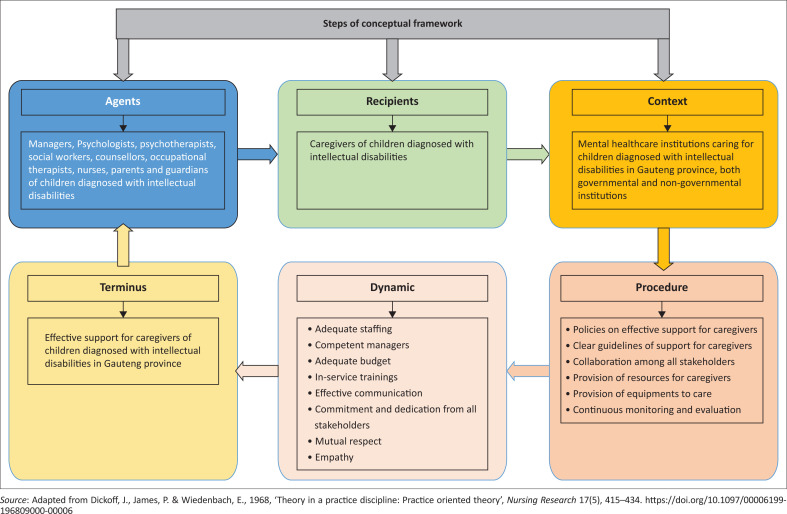
Conceptual framework of support for caregivers of children diagnosed with intellectual disabilities in the Gauteng province.

After classification of concepts (Figure 1), a conceptual framework of support for caregivers of children diagnosed with intellectual disabilities in the Gauteng province was finalised and mapped (Figure 2).

## Description of the conceptual framework

A conceptual framework of support for caregivers of children diagnosed with intellectual disabilities in the Gauteng province was developed using the six steps of Dickoff et al. (1968:422), as mapped in the grey shape of Figure 2. The bluish rectangular shape on the left-hand side is the agent, which is the first process of the framework. The second shape to follow is the terminus, which is a light greenish rectangular shape in the middle (Figure 2). The context follows on the right upper rectangular shape, coloured dark yellow. The dark pinkish rectangular shape on the right-hand side at the bottom represents the procedure of the framework, whereas the light pinkish shape in the middle is the dynamic aspect of the framework. The last shape situated on the left-hand corner at the bottom, which is light yellow, represents the terminus. All shapes are linked to each other with arrows to represent relationships between these processes.

## Relevancy of the conceptual framework

This conceptual framework is relevant to guide stakeholders on support for caregivers of children diagnosed with intellectual disabilities in the Gauteng province. Tomlinson (2013:58) affirms that in order to guide stakeholders, there is a need for development of an appropriate legal framework that provides support to caregivers across all domains. From the introductory statement, it has been noted that caregivers play an important role in providing care; therefore, they need to be supported at all times. The statement is further affirmed by Joseph and Joseph (2016:71), who say that caregivers need protection from all workplace hazards. The framework will therefore guide all stakeholders on support for caregivers of children diagnosed with intellectual disabilities in the Gauteng province. Furthermore, this framework will not only assist stakeholders of the Gauteng province but will also assist stakeholders of other provinces and could be utilised internationally.

## Assumptions of the conceptual framework

A conceptual framework is not merely a collection of concepts but rather a construct in which each concept plays an integral role (Jabareen 2009:51). According to Jabareen (2009:50), every concept has components and is defined by them; every concept has an irregular contour defined by its components; every concept contains ‘bits’ or components originating from other concepts; all concepts relate back to other concepts; every concept is considered as the point of coincidence, condensation or accumulation of its own components; and every concept must be understood relative to its own components, to other concepts, to the plane on which it is defined and to the problem it is supposed to resolve. It is against this background that the assumption of this conceptual framework is that each and every concept of this framework will add value to the outcome intended to be reached, which is ensuring effective support for caregivers of children diagnosed with intellectual disabilities in the Gauteng province.

## Components of the conceptual framework

### Agents

The question that was asked is: who are agents of this framework? Agents are who or what performs the activity, and these are people who will implement this framework (Dickoff et al. 1968:423). Findings of the study revealed that agents are managers, psychologists, psychotherapists, social workers, counsellors, occupational therapists, nurses, parents and guardians of children diagnosed with intellectual disabilities. Agents will ensure that the needs of caregivers are met. Their services and support will ensure that caregivers remain stress-free and experience no burnout related to caring for children diagnosed with intellectual disabilities. Managers are crucial in ensuring that caregivers are supported through requisition and allocation of adequate funds for purchasing of equipment necessary in provision of quality care for children, ensuring that caregivers are well remunerated, ensuring that caregivers are trained in intellectual disability, as well as ensuring that there are adequate staff in institutions catering for children diagnosed with intellectual disabilities, because without adequate staff, caregivers encounter heavy workloads and become overwhelmed due to difficulty in coping. Furthermore, managers must ensure that there are services such as debriefing and counselling for caregivers who feel overwhelmed because of caring. Managers need to improve communication with caregivers so that caregivers can be able to trust them and easily communicate with them regarding any issues affecting them and children. Managers must be approachable because if they are not, caregivers may be afraid to communicate their work-related frustrations with managers, hence becoming bottled-up, stressed and overwhelmed.

Mental healthcare practitioners such as psychologists, psychotherapists, social workers, counsellors, occupational therapists and nurses are also crucial, because when caregivers are stressed and overwhelmed, they are the relevant people who provide their services to caregivers, whether in the form of counselling, debriefing or pharmaceutical interventions where necessary. Furthermore, guardians and parents of children diagnosed with intellectual disabilities play an important role because without their support, caregivers feel overwhelmed. They need to take part in caring for their children through regular visitations, spending time with their children and having time-out with their children. In doing so, this will ease work pressure and lower stress levels that caregivers encounter. In addition, their visits will facilitate healing and mental easiness on children because children will see that they are not neglected but are loved and appreciated by their parents.

### Recipients

The question that was asked is: who are recipients of this framework? Recipients are who or what receives the activity (Dickoff et al. 1968:423). The recipients are caregivers of children diagnosed with intellectual disabilities in the Gauteng province because they will receive activities to be performed by agents of this framework. From the introductory statement, caring for children diagnosed with intellectual disabilities is challenging because it affects the psychological morbidity of caregivers and puts them at risk of poor mental and physical health. Caregivers experience emotional exhaustion, burnout, depersonalisation and personal difficulties associated with caring (Sallim et al. 2015:1036); hence, they need support. In supporting caregivers, challenges associated with caring will end. It is therefore necessary for agents of this framework to provide all the support that caregivers need.

### Context

The question that was asked is: in what context will the framework be implemented (Dickoff et al. 1968:423)? The context for this framework is all mental healthcare institutions caring for children diagnosed with intellectual disabilities in the Gauteng province, both governmental and non-governmental institutions. These institutions admit children diagnosed with intellectual disabilities; therefore, caregivers are the ones who have to take care of these children. Often times, parents and guardians bring children to these institutions and do not even visit them; thus, the work of caring solely becomes the responsibility of caregivers. It is therefore not surprising that caregivers become overwhelmed and stressed because of lack of support from even the parents of the children. Therefore, in support for caregivers, the relevant context is all institutions admitting children diagnosed with intellectual disabilities.

### Procedures

The question that was asked is: what are the guiding procedures, techniques, or protocols to implement this framework (Dickoff et al. 1968:423)?

The following are procedures of this framework: firstly, there must be policies on effective support for caregivers of children diagnosed with intellectual disabilities. Policies are a standard set of principles that guide a course of action. Public policies are established by the government, whereas private policies are created by organisations for institutional use. Many public policies are legally binding, meaning that individuals and institutions must comply with them (Keshia et al. 2018:97). Based on the statement by Keshia et al., if there are guiding principles that must be followed and adhered to regarding support for caregivers of children diagnosed with intellectual disabilities, then principles will be followed without any confusion, thus ensuring success of an outcome.

Secondly, there must be clear guidelines of support for caregivers. Guidelines help to avoid inefficiencies and optimise the value of healthcare expenditures by identifying practices that are unnecessary or unduly expensive. Furthermore, guidelines describe the quality of evidence and the degree of uncertainty that underlie the recommendations (Klein 2018:498). Therefore, all stakeholders must have clear guidelines regarding support for caregivers of children diagnosed with intellectual disabilities so as to assist in avoiding inefficiencies. Guidelines must address issues such as conduct of stakeholders towards caregivers, ordering or equipment, ensuring availability of resources, staff ratio to children and communication.

Thirdly, there must be collaboration among all stakeholders. According to Hugh et al. (2021:753), collaboration between healthcare, social services and other sectors is widely promoted as a route to improving population health, and it helps organisations to combine their skills and resources to meet individual, family and community needs. Therefore, collaboration among stakeholders will definitely assist to support caregivers, because where an individual institution is unable to provide services, perhaps because of budgetary constraints – for example, training of caregivers on intellectual disability and how to care for children with intellectual disability – another sector or service that is not financially constrained can easily come in and assist with training of caregivers.

Fourthly, there must be provision of adequate resources for caregivers to provide care. Resources must include material, financial and nonmaterial resources. Caregivers of children diagnosed with intellectual disabilities provide care to children who are fully dependent on them. Adequate staffing is very important to ease the workload of caring. Caregivers must be well remunerated and appreciated, and there must be equipment necessary to provide care, hence the importance of involving all stakeholders. Rivaz et al. (2017:IC02) confirm that to resolve challenges such as imbalanced workload, inappropriate nurse–patient ratios and inadequate physical resources, which negatively affect caregivers’ perceptions of the quality of caring practice environment, there must be improvement of the practice environment, with adequate staffing levels and appropriate allocation of physical resources.

Fifthly, there must be relevant equipment to provide care for caregivers. Looking at the difficulty of caring for a child diagnosed with intellectual disability, managers need to ensure that caregivers have the following necessary equipment to provide care: equipment to assist in lifting children, wheelchairs, supporting cushions, dippers and any other relevant equipment necessary to provide care. Having the necessary equipment reduces stress associated with caring, prevents physical harm to caregivers because they will not have to lift unnecessarily and improves their morale. Batt and Bathija (2018:1273) affirm that institutions must have sufficient budget for resources needed and to improve and maintain the health of caregivers.

There must be continuous monitoring and evaluation processes as emphasised by Abrahams (2019:a433). The results can illuminate, inform, make sense of a complex contextual environment and simplify complex situations and practices. Therefore, in order to ensure success of the intention of the framework, this process is necessary.

### Dynamics

The question that was asked is: what are the dynamics of this framework? Dynamics are power sources such as agents, recipients and context. Dynamics include activities to be performed to ensure success of a framework (Dickoff et al. 1968:431). The dynamics of this framework include adequate staffing, competent managers, adequate budget, in-service trainings, effective communication, commitment and dedication from all stakeholders, mutual respect and empathy. It is therefore clear that in order to ensure support for caregivers of children diagnosed with intellectual disabilities, there must be effective communication, commitment and dedication, mutual respect and empathy among all stakeholders. If all of these are practised, mental health institutions will be supported through adequate budget to ensure that there is adequate staffing, competent managers to run institutions and regular in-service trainings for caregivers, thus providing an improvement of the care received by children, as well as the promotion of the mental, physical, social, financial and total well-being of caregivers.

### Terminus

Terminus is the end product or outcome of the framework (Dickoff et al. 1968:423). The terminus for this framework is effective support for caregivers of children diagnosed with intellectual disabilities in the Gauteng province. Therefore, this framework will ensure that caregivers of children diagnosed with intellectual disabilities in the Gauteng province, across all mental health institutions catering for children with intellectual disabilities, are holistically supported, thus in turn promoting quality care for children.

### Limitations

In developing the framework, the results of the empirical phase were used. This phase had two stages: the systematic review as well as the qualitative research method. During the qualitative research method stage, semistructured interviews were conducted with both caregivers and mental healthcare professionals of one province of South Africa, the Gauteng province. As a result of the qualitative nature of the study, this framework cannot be generalised, but can be applied in other provinces of South Africa or internationally.

## Recommendations

The recommendations of this study are discussed under the headings of recommendations for practice, policy development and research.

### Recommendations for nursing practice

The lack of resources to provide care and ill treatment of caregivers by managers, as outlined by participants, are very concerning issues because they promote poor quality of healthcare delivery. It is therefore recommended that stakeholders within mental healthcare institutions take seriously all challenges and needs as outlined and bring changes within institutions by ensuring that all required resources, be they financial, human or material, become available for the provision of quality healthcare. Even though caregivers are theoretically acknowledged, there is still a gap in practical acknowledgement. The researcher therefore recommends that policymakers should do site visits to ensure that policies are well implemented by the managers of such institutions. Furthermore, there is a need for recognition of the role of caregivers in health and social departments. The researcher therefore recommends strengthening of such departments to ensure that caregivers are well recognised and appreciated.

### Suggestions for policy development

Policymakers need to acknowledge that caregivers of children diagnosed with intellectual disabilities have enormous challenges and needs that are not yet addressed. The researcher therefore recommends a review and evaluation of current health policies, as well as development of new policies that will address challenges and needs of caregivers of children diagnosed with intellectual disabilities.

### Advice for future research

Literature reviewed revealed no existing conceptual framework of support for caregivers of children diagnosed with intellectual disabilities in the Gauteng province, hence there is a need for development of a conceptual framework for caregivers in the province. The researchers therefore recommend future research studies that will utilise the framework to develop an effective support programme for caregivers of children diagnosed with intellectual disabilities in the Gauteng province. The development of a support programme will not only benefit caregivers, but it will improve quality of care for children diagnosed with intellectual disabilities. Furthermore, the researchers recommend future studies that will be conducted in other provinces and globally so that challenges and needs are better known and effectively addressed.

## Conclusion

The study was conducted to develop a conceptual framework of support for caregivers of children diagnosed with intellectual disabilities in the Gauteng province. The study adopted a qualitative, exploratory, descriptive and contextual research design. In this method, three phases were followed: an empirical phase, a classification of concepts and a development phase. Results of the empirical phase were used to classify concepts and to develop a conceptual framework, utilising the six steps of Dickoff et al. (1968:422). The developed framework serves as a guiding tool for future studies aiming at developing support programmes. The framework further guides stakeholders on how caregivers can effectively be supported so as to promote quality care services for children diagnosed with intellectual disabilities.
